# Two-year outcomes of the treat-and-extend regimen using aflibercept for treating diabetic macular oedema

**DOI:** 10.1038/s41598-020-78954-3

**Published:** 2020-12-16

**Authors:** Yu Cheol Kim, Jae Pil Shin, Kang Yeun Pak, Hyun Woong Kim, Min Sagong, Sang Joon Lee, In Young Chung, Sung Who Park, Ji Eun Lee

**Affiliations:** 1 Department of Ophthalmology, Keimyung University School of Medicine, Daegu, Korea; 2 Department of Ophthalmology, Kyungpook National University School of Medicine, Daegu, Korea; 3 Department of Ophthalmology, Haeundae Paik Hospital, Inje University College of Medicine, Busan, Korea; 4 Department of Ophthalmology, Yeungnam University College of Medicine, Daegu, Korea; 5 Department of Ophthalmology, Kosin University College of Medicine, Busan, Korea; 6 Department of Ophthalmology, Gyeongsang National University School of Medicine, Jinju, Korea; 7 Department of Ophthalmology, Pusan National University, School of Medicine, 49, Busandaehak-ro, Mulgeum-eup, Yangsan, Gyeongsangnam-do 50612 Korea; 8 Biomedical Institute, Pusan National University Hospital, Busan, Korea

**Keywords:** Diseases, Health care, Medical research

## Abstract

This study was performed to investigate the efficacy of the treat-and-extend regimen using aflibercept for treating diabetic macular oedema (DME). This prospective, multicentre, interventional, single-arm, 104-week clinical trial included 48 patients with DME visual impairment. The patients’ eyes received five consecutive intravitreal injections (2 mg aflibercept) every four weeks with two-week adjustments based on central subfield macular thickness (CSMT) changes. Injections were deferred when CSMT was stable. The number of injections, best-corrected visual acuity (BCVA), CSMT, and diabetic retinopathy severity scale scores were analysed. Compared to baseline, BCVA improved by + 9.1 letters at 52 weeks and was maintained with + 9.4-letter gain at 104 weeks (*P* < 0.001). Between baseline and 104 weeks, CSMT decreased from 489 to 298 μm (*P* < 0.001) and eyes with vision ≥ 20/40 increased from 17.4 to 43.5% (*P* = 0.007). The mean number of injections decreased from 8.5 in year one to 3.9 in year two. The injection interval was extended to ≥ 12 weeks in 56.5% of patients. The treat-and-extend regimen of aflibercept in DME showed 2-year efficacy comparable to that of fixed dosing regimens. The flexible dosing of this regimen reduced the number of injections in year two while maintaining efficacy.

## Introduction

Increased vascular permeability induced by vascular endothelial growth factor (VEGF) is believed to be one of the most important pathophysiological mechanism in diabetic macular oedema (DME). Accordingly, the efficacy of anti-VEGF injections in DME has been demonstrated in clinical trials as well as in real-world practice^1–4^. Despite the proven efficacy, monthly maintenance dosing is a tremendous burden for both patients and the healthcare system. Therefore, numerous efforts have been undertaken to develop a variable dosing regimen without losing the visual and anatomical gains expected with fixed dosing.

A treat-and-extend regimen (TER) is an individualised dosing scheme of titrating the injection interval based on the patient’s response^5^. The key advantage of TERs over *pro re nata* (PRN) regimens is a reduction in the numbers of visits and recurrences. In the era of the current pandemic, it is crucial to reduce the number of visits. Several clinical studies have showed favourable outcomes in treating DME using ranibizumab^6–8^. Aflibercept, another anti-VEGF agent, is more efficacious than ranibizumab in some subgroups of patients with DME^2,3^; however, information regarding TER outcomes in DME is scarce.

This study (treat-and-extend regimen using intra**VI**treal afli**B**ercept **I**n diabetic **M**acular edema [VIBIM] study) was designed to evaluate the efficacy of TER using aflibercept in DME. We previously published the results of the one-year interim analysis^9^, and present the 2-year outcomes are here.

## Methods

The study design has been described previously^9^. In brief, this prospective, multicentre, single-arm study (ClinicalTrials.gov ID: NCT02788877, date of registration: 02/June/2016) enrolled 48 eyes with DME at eight centres in South Korea. The study protocol was approved by the Institutional Review Board of Pusan National University Hospital and the other participating centres. All procedures were performed in the study according to the ethical standards of the institutional review board/ethics committee at each hospital and the tenets of the Declaration of Helsinki. Informed consent was obtained from all patients.

The key inclusion and exclusion criteria were comparable to those of the VISTA and VIVID studies^10^. Patients were eligible for enrolment if they met all of the following criteria: (1) age > 18 years; (2) diagnosis of centre-involving DME (central subfield macular thickness [CSMT] ≥ 300 μm on spectral-domain optical coherence tomography); (3) best-corrected visual acuity (BCVA) of 20/40 to 20/320 (Snellen visual acuity [VA]); (4) no history of laser photocoagulation in the study eye; (5) no anti-VEGF treatment in the study eye within 90 days prior to enrolment; and (6) no intra-/peri-ocular steroid injection in the study eye within 120 days prior to enrolment.

The TER algorithm of the VIBIM study have been previously described in detail^9^. The eyes received five consecutive intravitreal injections of 2 mg aflibercept every four weeks. The injection intervals were then adjusted by two weeks based on CSMT changes. When CSMT was < 250 μm and Snellen VA was 20/20 before receiving five injections, the loading injections were not necessarily completed. If CSMT worsened (increase ≥ 10%), stabilised (change < 10%), or improved (reduction ≥ 10%), the interval was shortened (minimum 4 weeks), extended (maximum 12 weeks), or maintained, respectively. If CSMT in the second year was stable over two consecutive 12-week interval visits, the injection was skipped, and the subsequent visit was scheduled 8 weeks later. If CSMT had not worsened after skipping injections, the patient was scheduled for observations without treatment at 8-week intervals. Starting from week 24, rescue treatments such as focal/grid laser treatment or intravitreal steroid injection were allowed at the physician’s discretion only in cases where CSMT increased up to 10% from baseline and VA decreased by > 10 Early Treatment Diabetic Retinopathy Study (ETDRS) chart letters. The visit and treatment schedules were automatically determined by submitting the BCVA and CSMT values to a web application.

On every visit after enrolment, BCVA tests (using ETDRS chart scores), slit-lamp examinations, intraocular pressure measurements using applanation tonometry, fundus photography, and spectral-domain optical coherence tomography were performed. At baseline, week 52, and week 104, fluorescein angiography images were obtained. To determine the severity of retinopathy, the diabetic retinopathy severity scale (DRSS) was evaluated according to the ETDRS scale and was graded as low (DRSS score ≤ 43), moderate (DRSS score = 47), or high (DRSS score ≥ 53) risk.

The primary efficacy endpoint was the change in BCVA as indicated by ETDRS letters from baseline to week 104. All statistical analyses were performed using SPSS v25 (IBM Corp., Armonk, NY, USA). The null hypothesis of no difference was rejected if *P*-value was < 0.05.

## Results

### Demographics and baseline characteristics

The study enrolled 48 patients, and 46 (23 men and 23 women, 59.4 ± 12.4 years of age) completed all the scheduled visits during the 104-week study period. All the patients had non-insulin-dependent diabetes mellitus. The demographic and baseline characteristics did not differ from those in the 1-year VIBIM report^9^. As previously reported, the mean duration since the diagnosis of diabetes was 16.6 ± 8.5 years. The severity of diabetic retinopathy at baseline included moderate non-proliferative diabetic retinopathy (NPDR) (29 eyes), severe NPDR (12 eyes), and proliferative diabetic retinopathy (5 eyes). The proportion of treatment-naive patients was 43.5% (*n* = 20) and the mean values of haemoglobin A1c, blood urea nitrogen, creatinine, low density lipoprotein, and triglycerides were 7.8 ± 1.5%, 19.7 ± 10.1 mg/dL, 1.1 ± 0.6 mg/dL, 90.5 ± 37.0 mg/dL, and 160.7 ± 89.0 mg/dL, respectively. The overall BCVA and CSMT values were 52.5 ± 19.7 (ETDRS letters) and 489.4 ± 130.4 μm, respectively.

### Treatment experience

The mean number of injections was 12.4 ± 3.1 (range 9–23; median: 13) over 2 years. The number of injections decreased substantially from 8.5 ± 0.8 (range 8–12; median: 8) in the first year to 3.9 ± 2.5 (range 1–11; median: 4) in the second year. At the last visit within the study period, 57% of the patients had injection interval of ≥ 12 weeks, including 19 patients (41%) with deferred injections (Table 1). Excluding the obligatory visits for assessments only, the patients visited the clinic 14.5 ± 1.9 (range 13–23, median: 14) times. No patients received rescue treatments during the 104-week study period.

**Table 1:** Number of patients receiving aflibercept injections at week 52 and 104 at the intervals specified.

Injection interval (weeks)	Week 52	Week 104
4	3 (6.5%)	1 (2.2%)
6	1 (2.2%)	3 (6.5%)
8	4 (8.7%)	3 (6.5%)
10	4 (8.7%)	13 (28.3%)
12	34 (73.9%)	7 (15.2%)
Deferred injection		19 (41.3%)

### Efficacy of the treatment regimen

**Figure 1:**
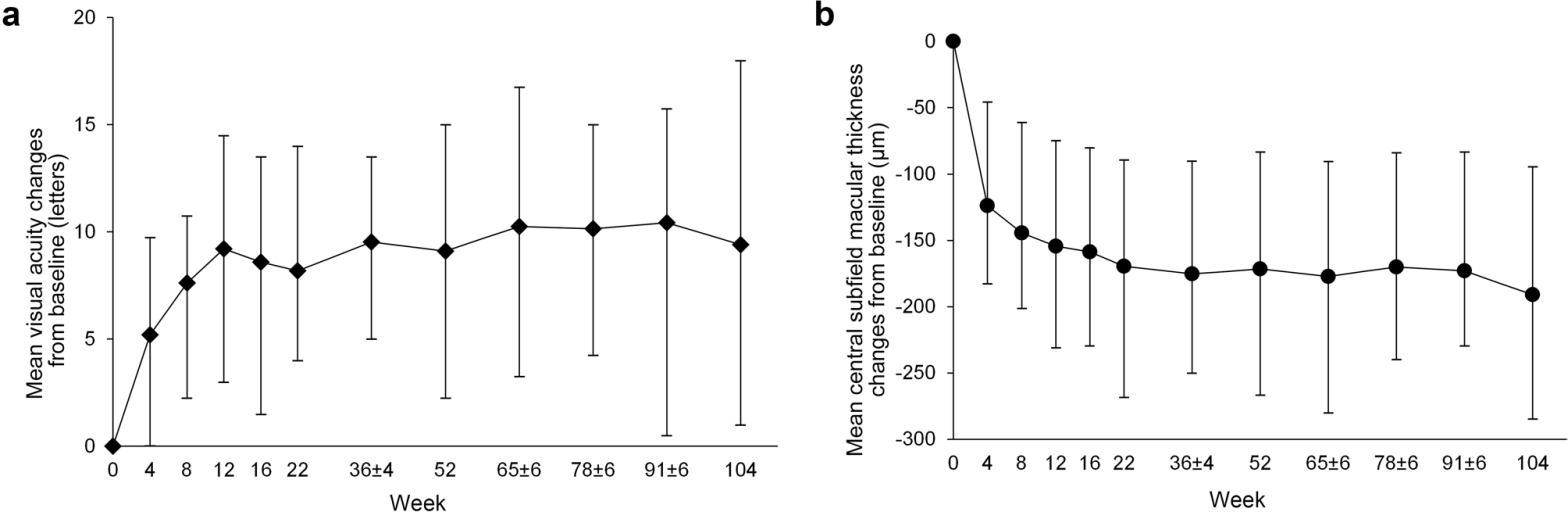
Best-corrected visual acuity (**a**) and central subfield macular thickness (**b**) changes from baseline to week 104. Error bars indicate interquartile ranges.

**Figure 2:**
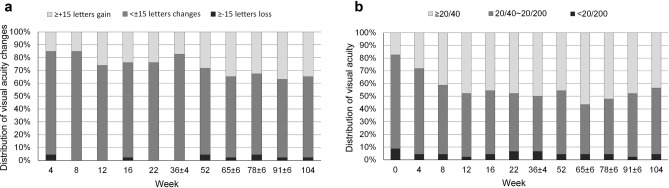
Distribution of visual acuity changes (**a**) and best-corrected visual acuity (**b**) from baseline to week 104.

A significant improvement in BCVA compared to baseline was noted beginning at week 4 (+ 5.0 ± 9.7 letters; *P* = 0.001) and continued throughout the study to weeks 52 and 104 (+ 9.1 and + 9.4 letters, respectively; both* P* < 0.001; Fig. 1a). CSMT decreased from 489.4 μm at baseline to 398.3 μm (-91.1 μm) at 1 year and to 298.3 μm (− 191.1 μm) at 2 years (Fig. 1b). The proportion of eyes that gained ≥ 15 letters was 28.3% at 1 year and 34.8% at 2 years (Fig. 2a). The percentage of eyes with BCVA ≥ 20/40 increased from 17.4% at baseline to 43.5% at week 104 (*P* = 0.013; Fig. 2b). Four patients (8.7%) achieved 20/20 vision at the final visit.

DRSS scores and their changes are shown in Table 2. At least a 2-step improvement was noted in 27.5% and 30.2% of patients at 1 and 2 years, respectively. In some patients, worsening of the diabetic state was noted during the second year. At 2 years, a ≥ 2-step aggravation was seen in 7.0% of patients compared with the DRSS score at baseline and in 12.5% compared with the score at 1 year.

**Table 2:** Changes in Diabetic Retinopathy Severity Scale (DRSS) scores from baseline to week 104.

DRSS score	Baseline	Week 52	Week 104
Gradable patients	*n* = 44	*n* = 42	*n* = 44
Low risk (DRSS ≤ 43)	18 (40.9%)	30 (71.4%)	28 (63.6%)
Moderate risk (DRSS = 47)	9 (20.5%)	5 (11.9%)	5 (11.4%)
High risk (DRSS ≥ 53)	17 (38.6%)	7 (16.7%)	11 (25.0%)

DRSS changes	Week 0–54	Week 0–104	Week 54–104
Improvement ≥ 2 steps	11 (27.5%)	13 (30.2%)	1 (2.5%)
Aggravation ≥ 2 steps	0 (0%)	3 (7.0%)	5 (12.5%)

### Subgroup analysis by various stratifications

**Figure 3:**
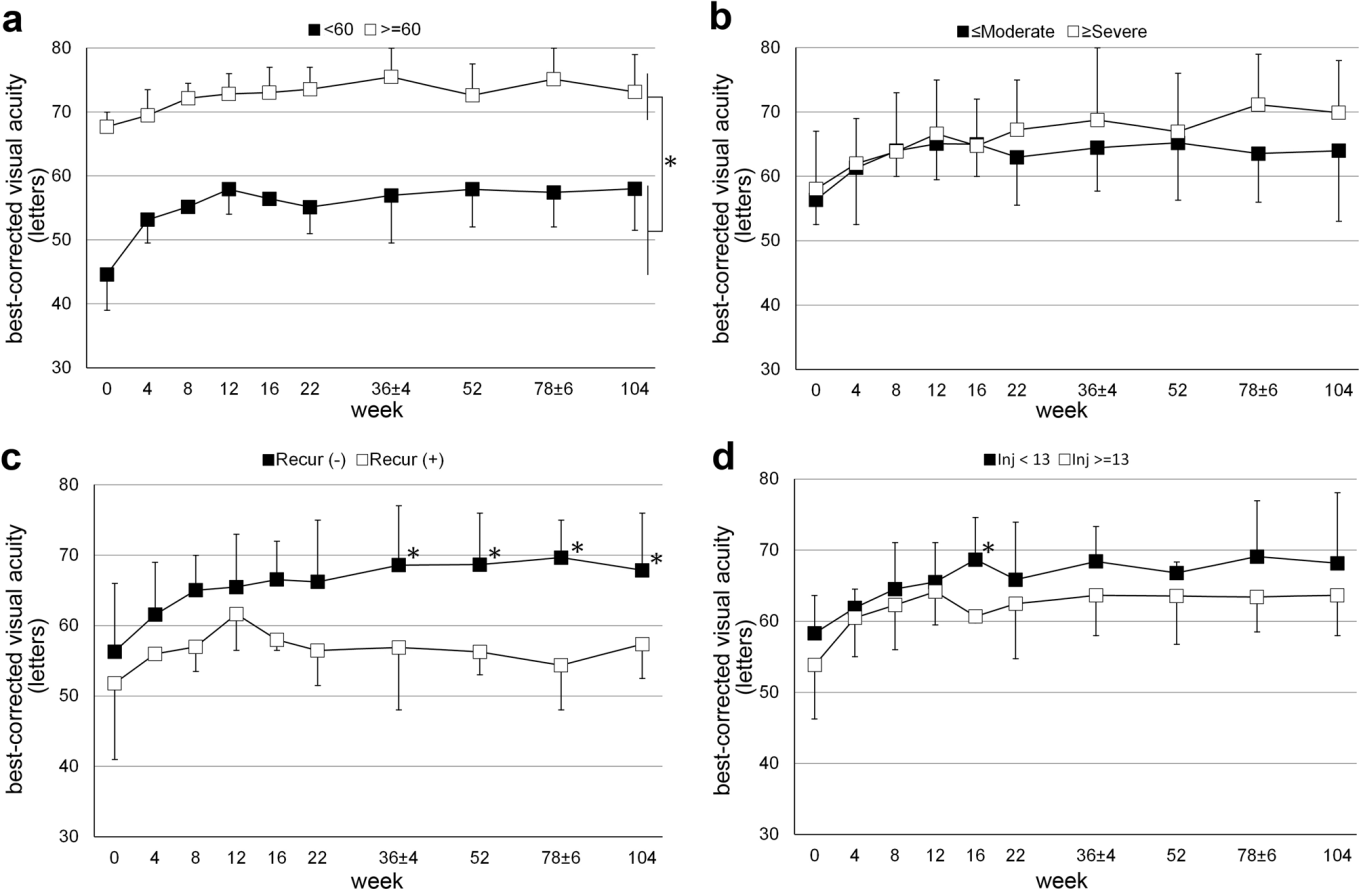
Visual acuity according to various stratifications including baseline visual acuity (**a**), baseline diabetic retinopathy severity scale (**b**), recurrence of macular oedema within the first year (**c**), and total number of injections in 2 years (**d**). *P < 0.05; Mann–Whitney-U test.

The visual outcomes were analysed using various stratifications according to the baseline visual acuity and DRSS score, recurrences in the first year, and total number of injections (Fig. 3). The patients with baseline BCVA worse than 60 letters had significantly more improvement than those with baseline BCVA of 60 letters or better. The difference between the two BCVA groups was statistically significant over the entire 2-year period (*P* < 0.05; Fig. 3a). Stratification by baseline DRSS score did not show a difference in BCVA for the study period (Fig. 3b). In the interim analysis, the group with episodes of worsened DME in the first year had significantly worse BCVA at the end of the year^9^. This difference was maintained in the second year (*P* < 0.05; Fig. 3c). Furthermore, no difference in BCVA was observed between the two groups divided according to the total number of injections within 2 years (Fig. 3d).

### Safety

An Anti-Platelet Trialists’ Collaboration event was not identified during the study period. Ten cases of severe adverse events were reported, including cataract in the study eye, cataract in the fellow eye, posterior capsular opacity, diabetic feet (two cases), hypertension, prostate cancer, vitamin B12 deficiency, cervical spine fracture, and ovarian tumour, which were not presumed to be related to the study drug or procedures.

## Discussion

Monthly or bimonthly fixed dosing anti-VEGF treatment is the most effective regimen for the treatment of neovascular age-related macular degeneration (nAMD) and DME. However, fixed dosing lacks long-term practicability in real-world settings due to overtreatment and high costs. Consequently, PRNs or TERs have been suggested as feasible alternatives^5^. Among nAMD treatments, TER is regarded as the most popular treatment regimen.

The VIBIM study investigated the efficacy of 2-year TER in the treatment of DME using aflibercept. The results showed that BCVA improved by a mean of 9.4 letters and gain ≥ 15 ETDRS letters in 34.8% patients with a mean of 12.4 ± 3.0 injections. The VA outcome in the present study was comparable to those of fixed dosing schemes (2 mg aflibercept every eight weeks: 2q8) in the VIVID (+ 9.4 letters) and VISTA (+ 11.1 letters) studies and TER with ranibizumab (+ 9.6 letters for TREX-DME)^8,11^. The proportions of patients with ≥ 15 letters gained were also comparable. However, the number of injections in the second year (3.9) was less than those in the other studies (4.9, 5.1, and 8.2 in VIVID, VISTA, and TREX-DME, respectively; Table 3)^8,11^. At the final visit in the current study, more than 40% of the patients deferred anti-VEGF injection for DME. These results substantiated that TER reduced DME overtreatment. The anatomical and functional improvements achieved in the loading phase were maintained for 2 years in the treat-and-extend phase.

**Table 3:** Comparison of the 2-year results between the current VIBIM study and previous studies.

Outcomes	VISTA^11^ 2q8	VIVID^11^ 2q8	VIBIM	RETAIN^7^	TREX-DME^8^	Protocol T^3^
Regimen	5 loadings + 2q8	5 loadings + 2q8	5 loadings + TER	3 loadings + TER	4 loadings + TER	6 loadings + PRN
Drug	Aflibercept 2.0 mg	Aflibercept 2.0 mg	Aflibercept 2.0 mg	Ranibizumab 0.5 mg	Ranibizumab 0.3 mg	Aflibercept 2.0 mg
Letter score changes	+ 11.1	+ 9.4	+ 9.4	+ 6.5	+ 9.6	+ 12.8
≥ 15-letter gain (%)	33.1	31.3	34.8	Not shown	Not shown	38.8
CSMT reduction (μm)	185.9	183.1	171.7	113.0	140.0	171.0
DRSS score ≥ 2-step improvement (%)	37.1	32.6	29.5	Not shown	Not shown	24.8
Injection numbers (over 2 years)	13.5	13.6	12.4	12.8	18.9	15 (median)
Injection numbers (in year 2)	5.1	4.9	3.9	n.a	8.2	5
Rescue laser (%)	8.6	11.1	0	0	0	41

2q8, a fixed dosing regimen of 2 mg every 8 weeks; TER, treat-and-extend regimen; PRN, *pro re nata*; CSMT, central subfield macular thickness; DRSS, diabetic retinopathy severity scale.

A ≥ 2-step improvement in DRSS score was noted in 27.5% and 30.2% of eyes at 1 and 2 years, respectively. In the 2q8 groups of VISTA and VIVID studies, a respective improvement of at least two steps in DRSS score was noted in 29.1% and 27.7% at week 52 and 37.1% and 32.6% at week 100, resepctively^4,10,11^. Although a ≥ 2-step improvement in DRSS score was observed in an additional 2.5% of the eyes in the second year of the current study, a ≥ 2-step aggravation in DRSS score was also noted in 12.5% of them. The proportion of aggravation ≥ 2 steps from baseline was 7%. Compared with VISTA and VIVID results, the DRSS scores in the present study are presumed to have been affected by the reduced number of injections. In Protocol S of the DRCR.net, approximately 60% of patients resumed injections within 16 weeks after 0.5 mg ranibizumab deferral, which suggests that anti-VEGF cannot suppress the progression of diabetic retinopathy for more than 4 months^12^. Accordingly, these results imply that the durability of anti-VEGF treatments differs between diabetic retinopathy and DME; therefore, diabetic retinopathy progression should be monitored carefully when the treatment intervals are extended with TER based on DME state.

It is noteworthy that in the current study, additional laser photocoagulation was not performed during the 2-year study period, whereas 41% of the eyes underwent at least one session of focal/grid laser photocoagulation during the 2 years of Protocol T^3^, and rescue laser treatment was provided to 8.6% and 11.1% of the 2q8 groups in the VISTA and VIVID studies, respectively, during their 100-week study periods^11^. Nevertheless, the final results of vision and CSMT were comparable among the four studies. In the TREX-DME study (injection of 0.3 mg ranibizumab q4w), TER without laser photocoagulation (TREX) and TER with angiography-guided laser photocoagulation (GILA) groups were compared. It was concluded that laser supplementation of treat-and-extend ranibizumab treatments in DME is of no added value^8^. In our study, four eyes (8.7%) had an injection interval of < 8 weeks at the end of the study; therefore, even 8-week fixed dosing would have been an undertreatment for these eyes and might have resulted in rescue treatments. Accordingly, TER tailors the treatment dosing to avoid not only overtreatment but also undertreatment; furthermore, it reduces the need for focal/grid laser rescue treatments in DME.

TERs are known for two advantages: one is their cost-effectiveness due to less frequent visits and the other is their increased efficacy based on proactive treatments. However, TER involves more injections than a PRN regimen, which may lead to overtreatment. The effects of fewer visits and more injections appear to offset each other, and the cost-effectiveness of a PRN regimen ($15,880.07/year in the PrONTO study) and TER ($16,114.52 and $13,971.44 for year one and two, respectively) are similar for nAMD treatments in the United States^13^. Although the VIBIM and TREX-DME studies showed that the efficacies of DME TERs were comparable to those of fixed dosing regimens, the possibility of overtreatment persists compared with a PRN regimen. In the RETAIN study, which used 0.5 mg ranibizumab, both the treat-and-extend laser group (12.4 injections) and the treat-and-extend non-laser group (12.8 injections) required more injections than the PRN group (10.7 injections) over 2 years^7^. Nonetheless, less frequent visits in TER would be an apparent merit over a PRN regimen as the pandemic of COVID-19 prevails. Accurate numbers of real visits have not been stated in previous studies, but the approximate numbers can be estimated based on the anti-VEGF and injection strategy, such as PRN and fixed dosing. In PRN, the number of scheduled visits will be 24–26 for 2 years. If a loading phase of monthly five injections is applied, the number of visits will be 15–16 and 24–26 in fixed dosing using aflibercept and ranibizumab, respectively. Although the number of visits for 2 years in our study (14.5) appears to be similar to that in fixed dosing, fewer number of injections in year two in our study (3.9) than those in VIVID (4.9) and VISTA (5.1) studies implies that the gap in the number of visits and injections between TER and fixed dosing regimen will increase on long-term follow-up. The TER algorithm in VIBIM study is modified from that of a typical TER by applying deferment of injection to prevent overtreatment since typical TER includes an injection at every visit. Accordingly, TER in this study included more visits (14.5) than the number of injections (12.4) and, possibly, fewer injections than those in typical TER, such as the aflibercept TER study in DME by Curry et al., which was 11.2 ± 1.56 (median, 11) in year one and 6.9 ± 3.2 (median, 6) in year two^14^.

Regarding the efficacy of proactive treatment in TERs, a comparison study between TERs and PRNs in nAMD proved the advantages of proactive treatments and revealed the superior efficacy of TERs over PRNs in BCVA and CSMT^15^. However, a similar comparison in DMEs has not yet been reported, and the necessity for proactive treatments is debated. Exudative changes in nAMD related to the proliferation of macular new vessels cause irreversible damage to the retinal cells at an early stage of the disease. The decrease in vision in DME usually originates from inner retinal oedema that affects Müller cells but precedes neuronal damage. This oedema disturbs light guidance and transmission to photoreceptors and can be reversed if treated promptly. Several prospective randomised studies have revealed that, compared with the baseline, the control group may attain vision comparable to the group that received anti-VEGF treatments even if anti-VEGF injections were delayed^16,17^. Due to the pathophysiological differences between nAMD and DME, proactive treatments may not be as valuable in DME as for nAMD. Comparison studies between DME PRNs and TERs will reveal the value of proactive DME treatments.

Among previous DME TER studies, the RETAIN study showed that TERs were non-inferior to PRN regimens^7^. The TREX-DME study revealed that TERs were comparable to fixed dosing regimens^8^. The results of the recent 2-year TER trial with aflibercept by Curry at el. were reported to be comparable to those of previous PRN regimens^14^. The retrospective comparison of 2-year outcomes between ranibizumab and aflibercept in DME with TER by Chujo et al. showed no significant differences in effectiveness between ranibizumab and aflibercept^18^. The 1-year prospective clinical trial of aflibercept TER in DME by Mieno et al. suggested aflibercept TER as an effective treatment option^19^. Compared with TER in the treatment of nAMD, the TER algorithm for DME is complicated and varies in the number of monthly loading injections as well as the standards determining whether the injection interval is extended, maintained, or shortened. The TREX-DME study had four loading injections; the RETAIN study had three loading doses; the study by Mieno et al. had two loading doses; and the VIBIM study had five loading doses, which is currently recommended in the anti-VEGF treatment in DME^7,8,10,19,20^. The standards of injection interval may be based on defined CSMT values or proportional changes in CSMT from the baseline or the previous visit. The differences in TER algorithms between DME clinical trials are larger than those in nAMD studies. Accordingly, the different outcomes of various clinical trials in DME with TER possibly arise from not only different drugs but also different TER algorithms. Although this VIBIM study has the limitation of a being a single-arm study with a relatively small number of patients, it is a multicentre, prospective 2-year DME TER study, which used aflibercept and highlighted comparable efficacy with fixed dosing regimens with fewer injections than previous PRN regimens and no rescue laser treatments. The superb outcomes are probably due to the unique TER algorithm including the initial intensive treatment (loading phase of five monthly injections), the strategy to avoid overtreatment (deferment of injections), and the injection-interval standards not being based on fixed CSMT.

Various stratifications were performed to find a prognostic biomarker in post-hoc analyses. The baseline visual acuity and DME recurrence in the first year were predictive of BCVA at 2 years. By contrast, the baseline DRSS score and the total number of injections did not have this predictive value. Recurrence, defined as an episode of DME worsening within the first year of the TER, was associated with decreased BCVAs at one and two years. As described in the 1-year report, there was no baseline difference between the two groups defined by recurrence^9^. Identifying additional biomarkers would, therefore, be required when choosing the optimal management plan for patients with DME.

In conclusion, the 2-year efficacies of the TER in DME using aflibercept in the current study were comparable to those of fixed dosing regimens in the pivotal trials. The flexible dosing of this TER reduced the number of intravitreal injections in the second year and avoided overtreatment without a reduction in the expected efficacy. However, aggravation of diabetic retinopathy was noted in some eyes, which was most likely related to the reduced number of injections.

## Data Availability

The datasets generated and/or analysed during the present study are available from the corresponding author on reasonable request.
